# Incretin receptor agonism rapidly inhibits AgRP neurons to suppress food intake in mice

**DOI:** 10.1172/JCI186652

**Published:** 2025-08-26

**Authors:** Hayley E. McMorrow, Andrew B. Cohen, Carolyn M. Lorch, Nikolas W. Hayes, Stefan W. Fleps, Joshua A. Frydman, Jessica L. Xia, Ricardo J. Samms, Lisa R. Beutler

**Affiliations:** 1 Department of Medicine, Division of Endocrinology, Metabolism and Molecular Medicine,; 2 Interdepartmental Neuroscience Graduate Program, and; 3 Driskill Graduate Program in Life Sciences, Northwestern University, Chicago, Illinois, USA.; 4 Department of Neuroscience, Northwestern University, Chicago, Illinois, USA.; 5 Diabetes, Obesity and Complications Therapeutic Area, Eli Lilly, Indianapolis, Indiana, USA.

**Keywords:** Endocrinology, Neuroscience, Homeostasis, Obesity

## Abstract

The incretin receptor agonists semaglutide and tirzepatide have transformed the medical management of obesity. The neural mechanisms by which incretin analogs regulate appetite remain incompletely understood, and dissecting this process is critical for the development of next-generation antiobesity drugs that are more targeted and tolerable. Moreover, the physiologic functions of incretins in appetite regulation and gut-brain communication have remained elusive. Using in vivo fiber photometry, we discovered distinct pharmacologic and physiologic roles for the incretin hormones glucose-dependent insulinotropic peptide (GIP) and glucagon-like peptide-1 (GLP-1). We showed that GIP, but not GLP-1, was required for normal nutrient-mediated inhibition of hunger-promoting AgRP neurons. By contrast, both GIP and GLP-1 analogs at pharmacologic doses were sufficient to inhibit AgRP neurons. The magnitude of neural inhibition was proportional to the effect of each incretin on food intake, and dual GIP and GLP-1 receptor agonism more potently inhibited AgRP neurons and suppressed food intake than either agonist alone. Our results have revealed a role for endogenous GIP in gut-brain appetite regulation and indicate that incretin analogs act in part via AgRP neurons to mediate their anorectic effects.

## Abstract 2

Toward understanding incretin biology, we show that both GLP-1 and GIP inhibit hunger-promoting AgRP neurons, but only GIP is necessary for nutrient-mediated AgRP neuron inhibition.

## Graphical abstract

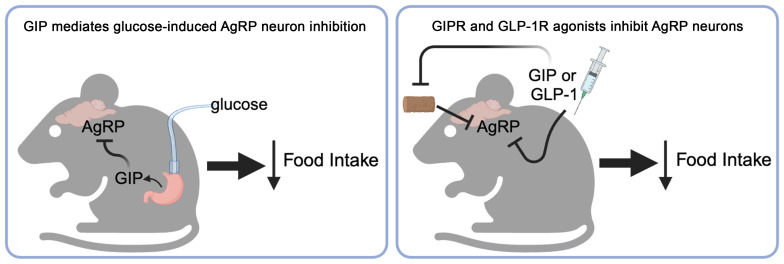

## Introduction

Analogs of the incretin hormones glucagon-like peptide-1 (GLP-1) and glucose-dependent insulinotropic peptide (GIP) have become mainstays of obesity and diabetes management. However, both the physiologic role of incretin hormones in the control of appetite and the pharmacologic mechanisms by which incretin-mimetic drugs suppress caloric intake remain incompletely understood.

Hunger-promoting AgRP-expressing neurons are an important hypothalamic population that regulates appetite. AgRP neuron activity is sufficient to promote feeding and critical for maintaining energy homeostasis, particularly after prolonged fasting (1–5). The dynamics of AgRP neurons are regulated by external sensory stimuli and interoceptive signals from the gastrointestinal tract to promote adaptive feeding behavior (6–10). Through macronutrient-dependent mechanisms, ingested nutrients inhibit AgRP neurons via multiple gut-derived signals and neural circuits (7, 11–15). Specifically, while it is known that cholecystokinin (CCK) is required for dietary fat-induced AgRP neuron inhibition (7), the molecular mediators of glucose-induced AgRP neuron inhibition remain unknown.

Here, we set out to investigate the effects of incretin hormones on in vivo AgRP neuron dynamics. Using fiber photometry, we found that GIP but not GLP-1 is necessary for nutrient-mediated AgRP neuron inhibition. To our knowledge, this represents a previously unknown role for endogenous GIP in controlling hunger and maintaining energy balance. By contrast, pharmacologic activation of both the GIP receptor (GIPR) and GLP-1 receptor (GLP-1R) acutely inhibit AgRP neurons in fasted mice and reduce the response of AgRP neurons to food. These effects appear to be additive, suggesting neural inhibition by GIPR versus GLP-1R agonism occurs via distinct mechanisms. Moreover, optogenetic stimulation of AgRP neurons partially attenuates incretin-induced feeding suppression, indicating that inhibiting AgRP neurons may be necessary for the full appetite-suppressing effects of incretin-based therapeutics. Taken together, our findings reveal neural mechanisms underlying the efficacy of incretin-mimetic obesity therapies. Understanding these drugs’ mechanisms of action is crucial for the development of next-generation obesity pharmacotherapies with improved tolerability.

## Results

### GIPR but not GLP-1R signaling is involved in glucose-mediated AgRP neuron inhibition.

**Figure 1:**
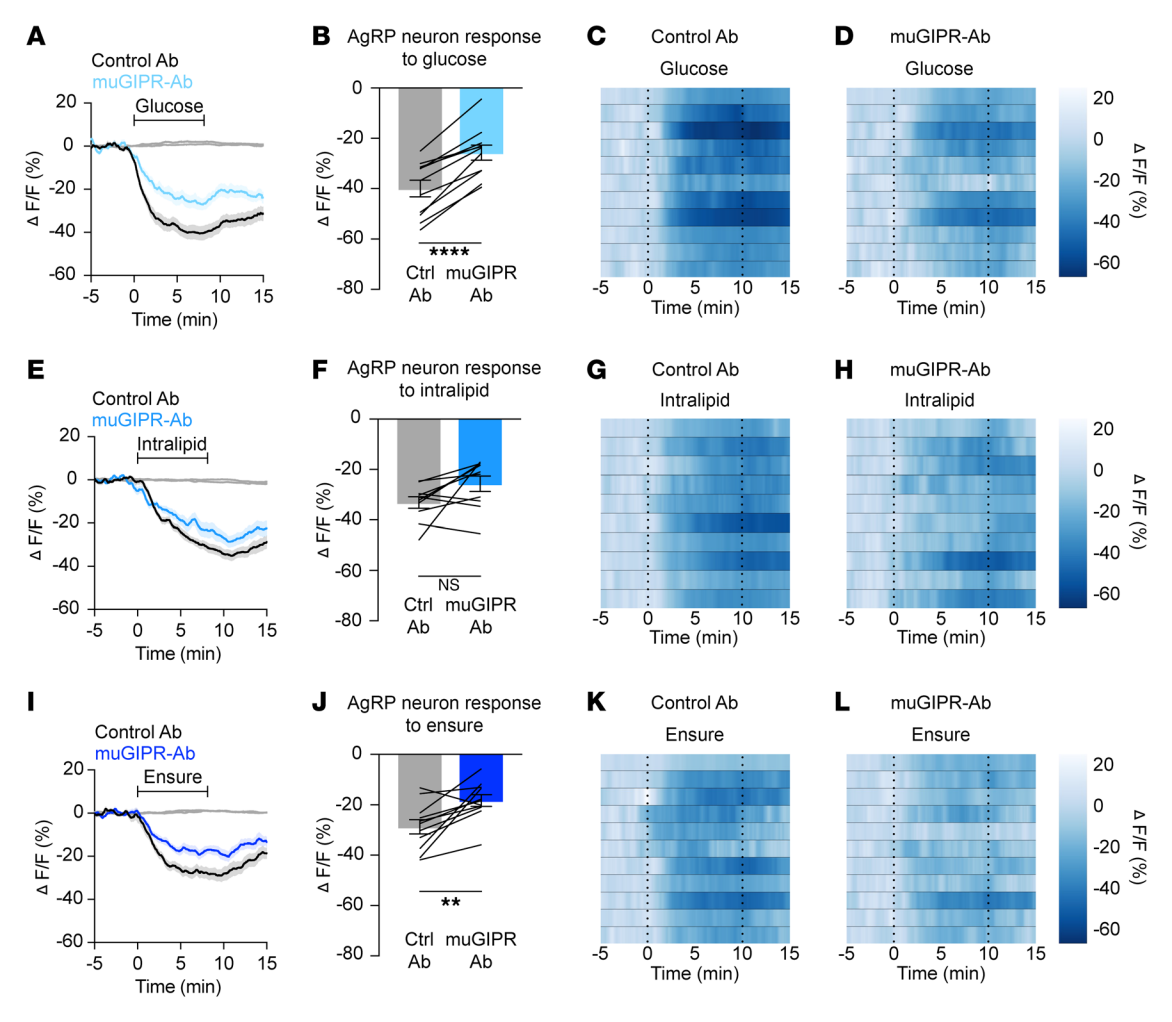
GIPR blockade partially attenuates nutrient-mediated AgRP neuron inhibition. (**A**, **E**, and **I**) Calcium signal in AgRP neurons from fasted mice during infusion of glucose (**A**), intralipid (**E**), or Ensure (**I**) after pretreatment with control or muGIPR-Ab as indicated. *n* = 10–11 mice per group. (**B**, **F**, and **J**) Average ΔF/F in mice from **A**, **E**, and **I** at the end of nutrient infusion. (**B**) paired *t* test, *P* < 0.0001; (**F**) paired *t* test, *P* = 0.0557; (**J**) paired *t* test, *P* = 0.0061. (**C**, **D**, **G**, **H**, **K**, and **L**) Heat maps showing ΔF/F in individual mice from **A**, **E**, and **I** during nutrient infusion. (**A**, **E**, and **I**) Isosbestic traces for all recordings are shown in gray. (**C**, **D**, **G**, **H**, **K**, and **L**) Vertical dashed lines indicate the start and end of nutrient infusions. (**B**, **F**, and **J**) Lines represent individual mice. Error bars indicate mean ± SEM. *t* tests: **P < 0.01, ****P < 0.0001.

**Figure 2:**
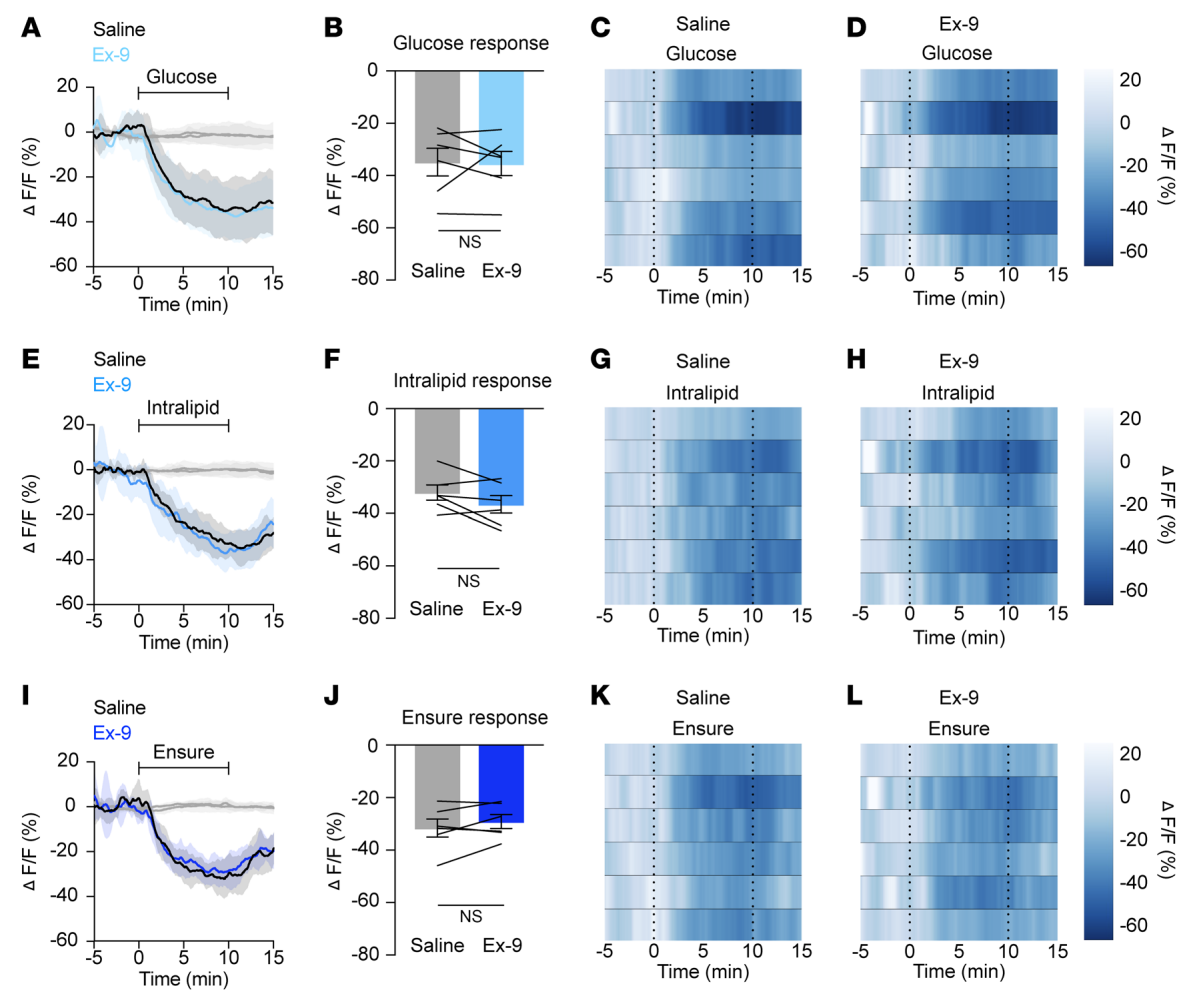
Signaling through GLP-1R is not necessary for nutrient-mediated AgRP neuron inhibition. (**A**, **E**, and **I**) Calcium signal in AgRP neurons from fasted mice during infusion of glucose (**A**), intralipid (**E**), or Ensure (**I**) after pretreatment with saline or Ex-9 as indicated. *n* = 6 mice per group. (**B**, **F**, and **J**) Average ΔF/F in mice from **A**, **E**, and **I** at the end of nutrient infusion. (**B**) paired *t* test, *P* = 0.8918; (**F**) paired *t* test, *P* = 0.1314; (**J**) paired *t* test, *P* = 0.2401). (**C**, **D**, **G**, **H**, **K**, and **L**) Heat maps showing ΔF/F in individual mice from **A**, **E**, and **I** during nutrient infusion. (**A**, **E**, and **I**) Isosbestic traces for all recordings are shown in gray. (**C**, **D**, **G**, **H**, **K**, and **L**) Vertical dashed lines indicate the start and end of nutrient infusions. (**B**, **F**, and **J**) Lines represent individual mice. Error bars indicate mean ± SEM.

To evaluate whether incretin hormones are necessary for nutrient-mediated AgRP neuron inhibition, we equipped mice for in vivo imaging of AgRP neurons using fiber photometry and intragastric nutrient infusion (7). Neural responses to nutrients were then measured in the presence versus absence of incretin receptor blockade. To examine the role of GIPR, we first pretreated mice with a control (nonneutralizing) antibody, then intragastrically administered glucose, lipid, or Ensure on different days. Following intragastric nutrient infusions under control conditions, mice were treated with a long-acting, neutralizing monoclonal murine GIPR blocking antibody (muGIPR-Ab) (16), and nutrient infusions were repeated. GIPR blockade significantly attenuated glucose- and Ensure-mediated AgRP neuron inhibition, but not lipid-induced AgRP neuron inhibition (Figure 1). Because muGIPR-Ab is long-acting, the order of pretreatments could not be counterbalanced. However, control experiments showed that mice maintained consistent neural responses to repeated intragastric nutrient infusions over 1 to 2 weeks in the absence of antibody treatment (Supplemental Figure 1; supplemental material available online with this article; https://doi.org/10.1172/JCI186652DS1), and multiple prior studies have shown consistent nutrient-mediated AgRP neural responses for several weeks in control mice (17–19). In contrast with the effect of GIPR blockade, pretreatment with the GLP-1R antagonist exendin (9-39) (Ex-9) had no effect on nutrient-mediated AgRP neuron inhibition (Figure 2).

In addition to their regulation by nutrient uptake in the gastrointestinal epithelium (12, 19), AgRP neurons are rapidly inhibited upon food presentation, prior to ingestion (6, 9, 10). The magnitude of this preconsummatory inhibition correlates with imminent food intake, which is required to sustain AgRP neuron inhibition (7). Neither muGIPR-Ab nor Ex-9 immediately impacted AgRP neuron inhibition in response to food presentation (Supplemental Figure 2, A, B, E, and F). However, both compounds slightly attenuated AgRP neuron inhibition 10 minutes after food exposure (Supplemental Figure 2, A, C, E, and G), at which point postingestive nutrient effects are likely contributing to AgRP neuron dynamics. The lack of an immediate effect on chow-induced AgRP neuron inhibition suggests that the blunted responses to gastrointestinal nutrients following muGIPR-Ab (Figure 1) are not likely due to a floor effect in the setting of altered baseline AgRP neuron activity. The delayed attenuation of chow-induced AgRP neuron inhibition following muGIPR-Ab is consistent with its effects on intragastric nutrient-mediated AgRP neuron inhibition (Figure 1). Pretreatment with muGIPR-Ab or Ex-9 did not alter acute fasting-induced food intake in WT mice (Supplemental Figure 2, D and H) in agreement with multiple reports demonstrating that the magnitude of rapid, preingestive AgRP neuron inhibition correlates with subsequent food intake (6, 7, 9, 17, 19). Finally, neither muGIPR-ab nor Ex-9 impacted AgRP neuron dynamics during an intragastric infusion of water (Supplemental Figure 3). Taken together, we have shown that GIP partially mediates glucose-dependent AgRP neuron inhibition. This may, in part, underlie the enhanced weight loss efficacy of dual GIP and GLP-1R agonists when compared with GLP-1R monoagonists, as GIPR activation may recapitulate the postingestive effects of glucose to reduce subsequent food intake.

### GIPR and GLP-1R analogs acutely inhibit AgRP neurons.

**Figure 3:**
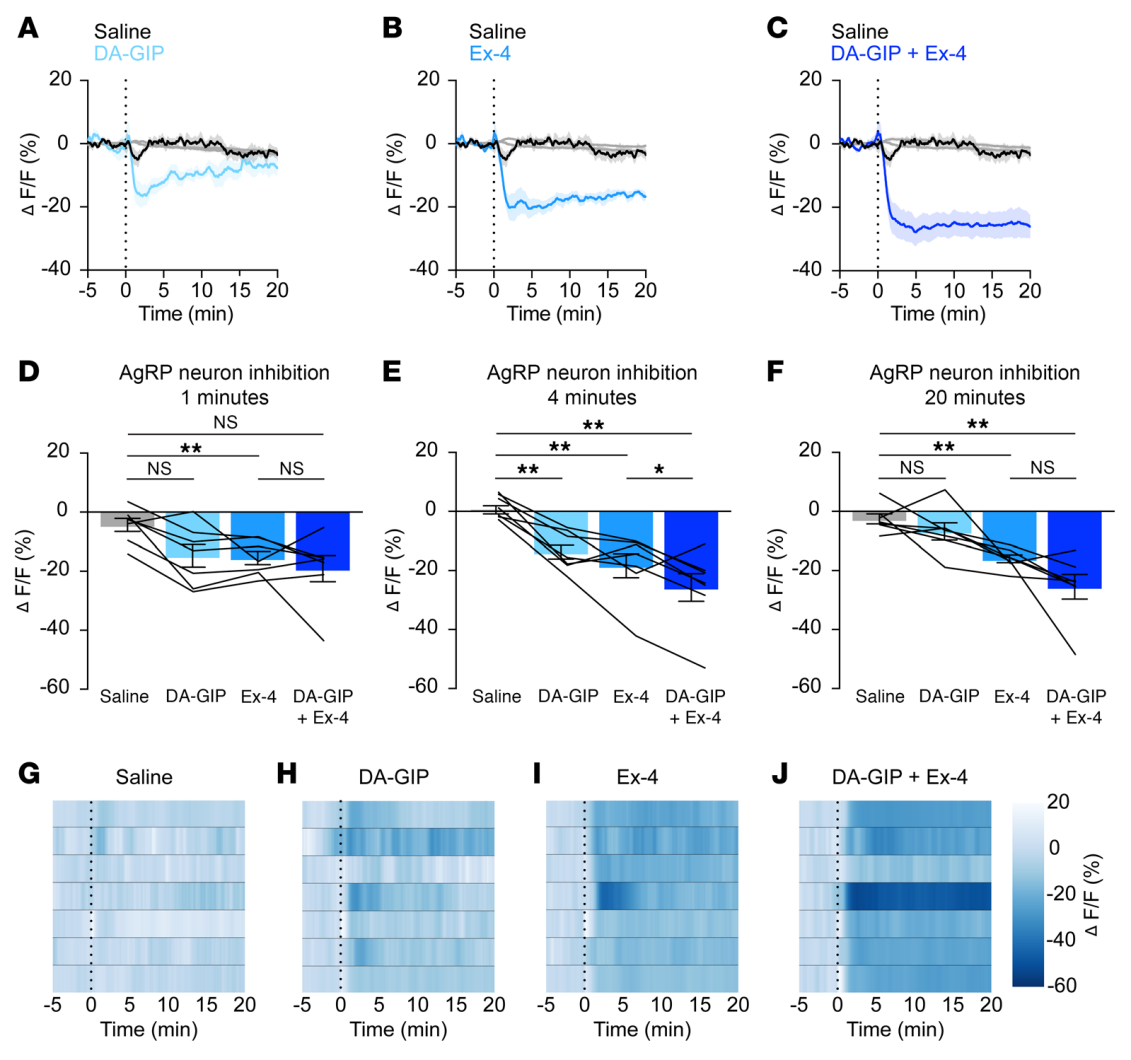
GIPR and GLP-1R agonists acutely inhibit AgRP neurons. (**A**–**C**) Calcium signal in AgRP neurons from fasted mice injected with DA-GIP (1 mg/kg; **A**), Ex-4 (1 mg/kg; **B**), or DA-GIP and Ex-4 (**C**) compared with saline as indicated. *n* = 7 mice per group. (**D**, **E**, and **F**) Average ΔF/F in mice from (**A**–**C**) 1 minute (**D**), 4 minutes (**E**), and 20 minutes (**F**) after injection. (**D**) 1-way ANOVA, *P* = 0.0232; (**E**) 1-way ANOVA, *P* < 0.0001; (**F**) 1-way ANOVA, *P* = 0.0003. (**G**–**J**) Heat maps showing ΔF/F in individual mice from (**A**–**C**) injected with saline (**G**), DA-GIP (**H**), Ex-4 (**I**), or DA-GIP and Ex-4 (**J**). (**A**–**C**) Isosbestic traces for all recordings are shown in gray. (**A**–**C**, and **G**–**J**) Vertical dashed lines indicate the time of injection. (**D**–**F**) Lines represent individual mice. Error bars indicate mean ± SEM. Post-hoc comparisons: *P < 0.05, **P < 0.01.

These experiments showed that GIPR but not GLP-1R is necessary for nutrient-mediated AgRP neuron inhibition. We next sought to examine whether pharmacologic doses of incretin analogs are sufficient to inhibit AgRP neurons in freely behaving mice using fiber photometry. Intraperitoneal (IP) injection of the GIP analog (D-Ala^2^)-GIP (DA-GIP) rapidly inhibited AgRP neurons (Figure 3, A and H), in agreement with its physiologic role in nutrient-mediated AgRP neuron inhibition (Figure 1). Surprisingly, while GLP-1R signaling is not necessary for nutrient-mediated AgRP neuron inhibition (Figure 2), the rapid acting GLP-1 analog Exendin-4 (Ex-4) was sufficient to inhibit AgRP neuron activity, consistent with prior ex vivo studies (Figure 3, B and I) (20, 21). Of note, our prior work showed that the GLP-1R agonist liraglutide does not rapidly modulate AgRP neuron activity in vivo (7). This is likely because liraglutide is more slowly absorbed and albumin-bound than Ex-4 and, thus, changes in neural activity are not expected on the relatively short time scale of fiber photometry recordings (22). The response of AgRP neurons to individual incretin analogs was dose dependent (Supplemental Figure 4, A–H, and Supplemental Figure 5, A–H). At a maximally effective dose of both agonists (1 mg/kg), Ex-4 induced greater neural inhibition than DA-GIP, and AgRP neuron inhibition in response to the combination of Ex-4 and DA-GIP was stronger than the response to Ex-4 alone (Figure 3).

### AgRP neuron stimulation partially rescues incretin-induced anorexia.

**Figure 4:**
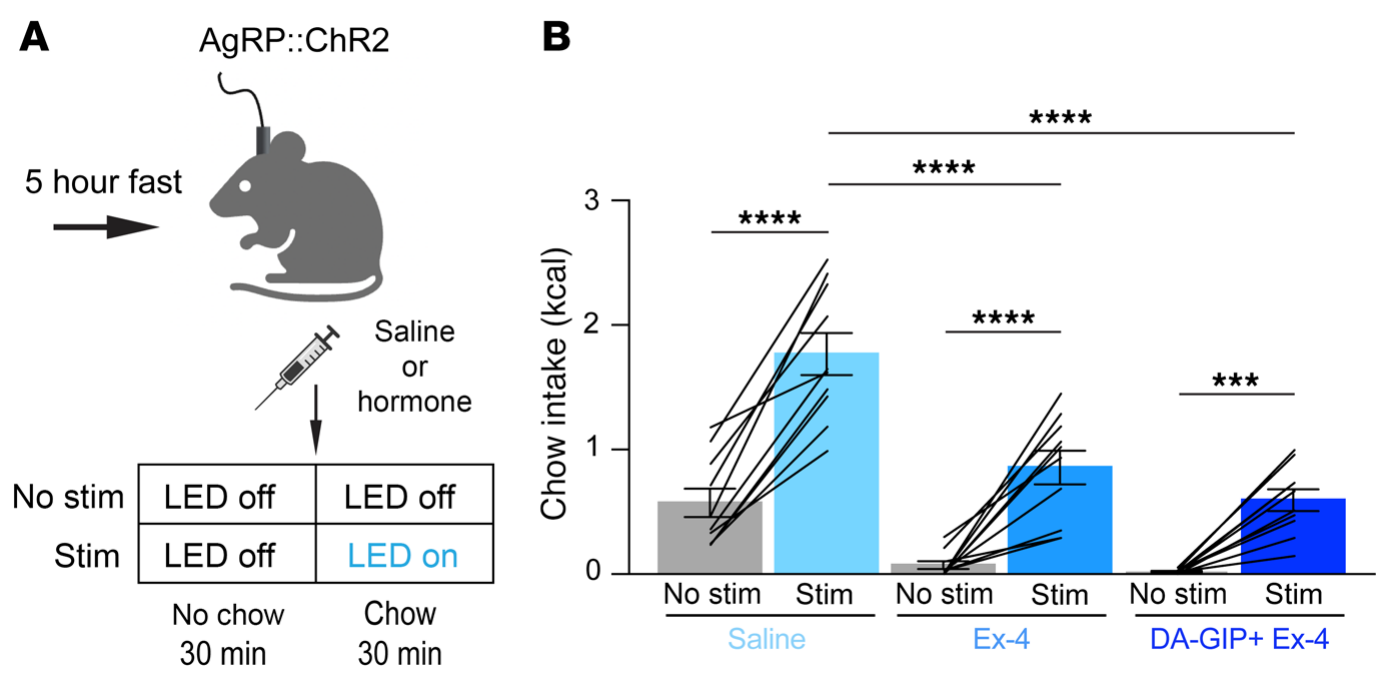
AgRP neuron stimulation partially rescues acute incretin-induced feeding suppression. (**A**) Experimental schematic. (**B**) 30-minute chow intake in fasted mice following injection of saline, Ex-4 (0.02 mg/kg), or DA-GIP (1 mg/kg), and Ex-4 (0.02 mg/kg) in the presence or absence of AgRP neuron stimulation as indicated. *n* = 10 mice per group. 2-way ANOVA, main effect of hormone treatment *P* < 0.0001, main effect of no stimulation versus stimulation *P* < 0.0001, interaction, *P* = 0.0036. Lines represent individual mice. Error bars indicate mean ± SEM. Post-hoc comparisons: ***P < 0.001, ****P < 0.0001.

We next used an optogenetic approach to investigate the behavioral relevance of incretin-mediated AgRP neuron inhibition. To determine whether AgRP neuron stimulation can overcome incretin receptor agonist-induced feeding suppression, mice that express channelrhodopsin-2 (ChR2) selectively in AgRP neurons (AgRP:ChR2 mice) were equipped for optogenetic stimulation of AgRP neuron cell bodies. These mice were fasted for 5 hours, habituated to feeding chambers for 30 minutes, then systemically treated with saline, Ex-4, or Ex-4 + DA-GIP and immediately refed in the absence or presence of light stimulation (Figure 4A). Due to the very subtle effect of GIPR monoagonism on acute food intake, we did not examine the effect of light stimulation on feeding following treatment with DA-GIP alone. In saline-treated mice, AgRP neuron stimulation significantly increased food intake, as expected (Figure 4B). AgRP neuron stimulation partially rescued the anorexia induced by both Ex-4 and Ex-4 + DA-GIP (Figure 4B). Thus, AgRP neuron inhibition likely contributes to incretin analog-induced appetite suppression, though future studies will be required to show causality and exclude the possibility that AgRP neuron stimulation induces food intake via a mechanism independent from incretin-induced anorexia.

### GIPR and GLP-1R analogs blunt AgRP neuron response to food presentation.

**Figure 5:**
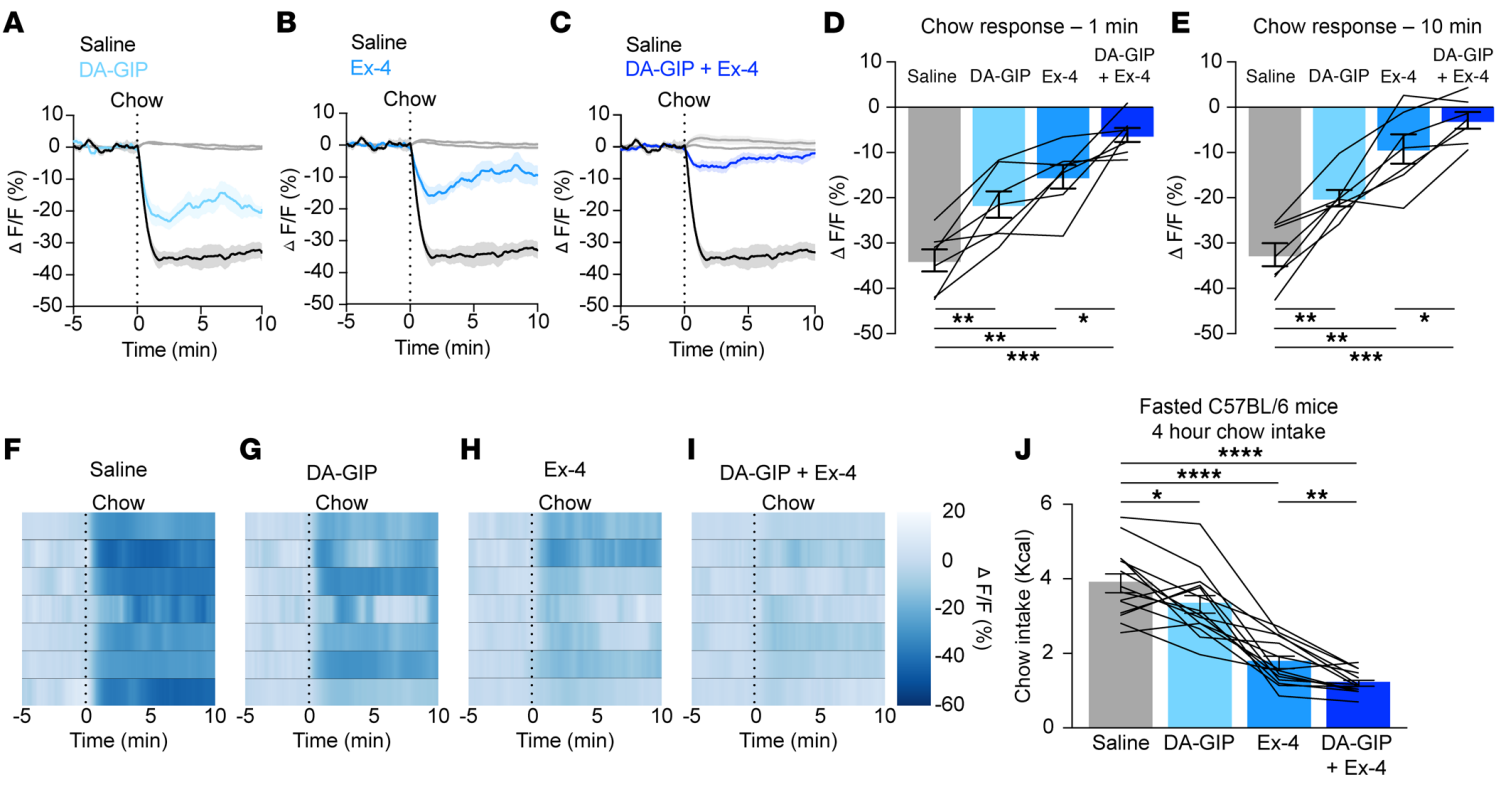
GIPR and GLP-1R agonists attenuate the AgRP neuron response to food presentation in proportion to their effect on food intake. (**A**–**C**) Calcium signal in AgRP neurons from fasted mice presented with chow 20 minutes after pretreatment with DA-GIP (1 mg/kg; **A**), Ex-4 (1 mg/kg; **B**), or DA-GIP and Ex-4 (**C**) compared with saline as indicated. *n* = 7 mice per group. (**D** and **E**) Average ΔF/F in mice from (**A**–**C**) 1 minute (**D**) and 10 minutes (**E**) after chow presentation. (**D**) 1-way ANOVA, *P* < 0.0001; (**E**) one-way ANOVA, *P* = 0.0001. (**F**–**I**) Heat maps showing ΔF/F in individual mice from **A**–**C** after chow presentation. (**J**) 4-hour chow intake following a 5-hour fast and incretin or saline injection as indicated in C57BL/6 mice. *n* = 14 mice per group. 1-way ANOVA, *P* < 0.0001. (**A**–**C**) Isosbestic traces for all recordings are shown in gray. (**A**–**C** and **F**–**I**) Vertical dashed lines indicate the time of chow presentation. (**D**, **E**, and **J**) Lines represent individual mice. Error bars indicate mean ± SEM. Post-hoc comparisons: *P < 0.05, **P < 0.01, ***P < 0.001, ****P < 0.0001.

As noted above, chow presentation induces rapid, preconsummatory AgRP neuron inhibition with a magnitude of inhibition that correlates with the quantity of subsequent food intake (6–10). As previously shown for the long-acting GLP-1R agonist liraglutide (20), pretreatment with DA-GIP or Ex-4 blunted subsequent chow-induced AgRP neuron inhibition compared to pretreatment with saline in the same mice (Figure 5, A–I). This blunted response persisted even at 10 minutes, after the start of ingestion (Figure 5E), reflecting incretin-induced reduction in food intake. While incretin agonism dramatically attenuated the AgRP neuron response to chow, it did not significantly alter the AgRP neuron response to intragastric Ensure infusion (Supplemental Figure 6). This is consistent with prior findings that, across a range of conditions, AgRP neuron response to food presentation correlates with subsequent food intake, whereas the neural response to gastric nutrients accurately reflects the quantity of nutrients consumed (7). Remarkably, when given in combination, Ex-4 and DA-GIP suppressed chow-induced neuron inhibition more than Ex-4 alone (Figure 5, B–E). Reduced AgRP neuron responses to chow presentation correlated with the feeding suppression induced by DA-GIP, Ex-4, or DA-GIP + Ex-4 in fasted WT mice (Figure 5J). Specifically, consistent with prior findings, acute treatment with DA-GIP modestly inhibited fast refeeding and significantly potentiated the suppression of food intake induced by Ex-4 (23). The effect of Ex-4 on chow-induced AgRP neuron inhibition was dose dependent (Supplemental Figure 5, I–P), consistent with dose-dependent effects of GLP-1 analogs on food intake (24). By contrast, the effect of DA-GIP on chow-induced AgRP neuron inhibition did not vary significantly with dose (Supplemental Figure 4, I–P), in line with the more subtle, acute effects of even high dose DA-GIP on food intake. Taken together, the increased effect of simultaneous GIPR and GLP-1R agonism relative to GLP-1R monoagonism on AgRP neuron dynamics aligns with mounting evidence for the superior efficacy of combined GIP and GLP-1R activation for the treatment of obesity (25–28), and may represent a partial mechanism for the remarkable weight loss induced by dual incretin agonism.

### Diet-induced obesity does not alter incretin-mediated AgRP neuron inhibition.

**Figure 6:**
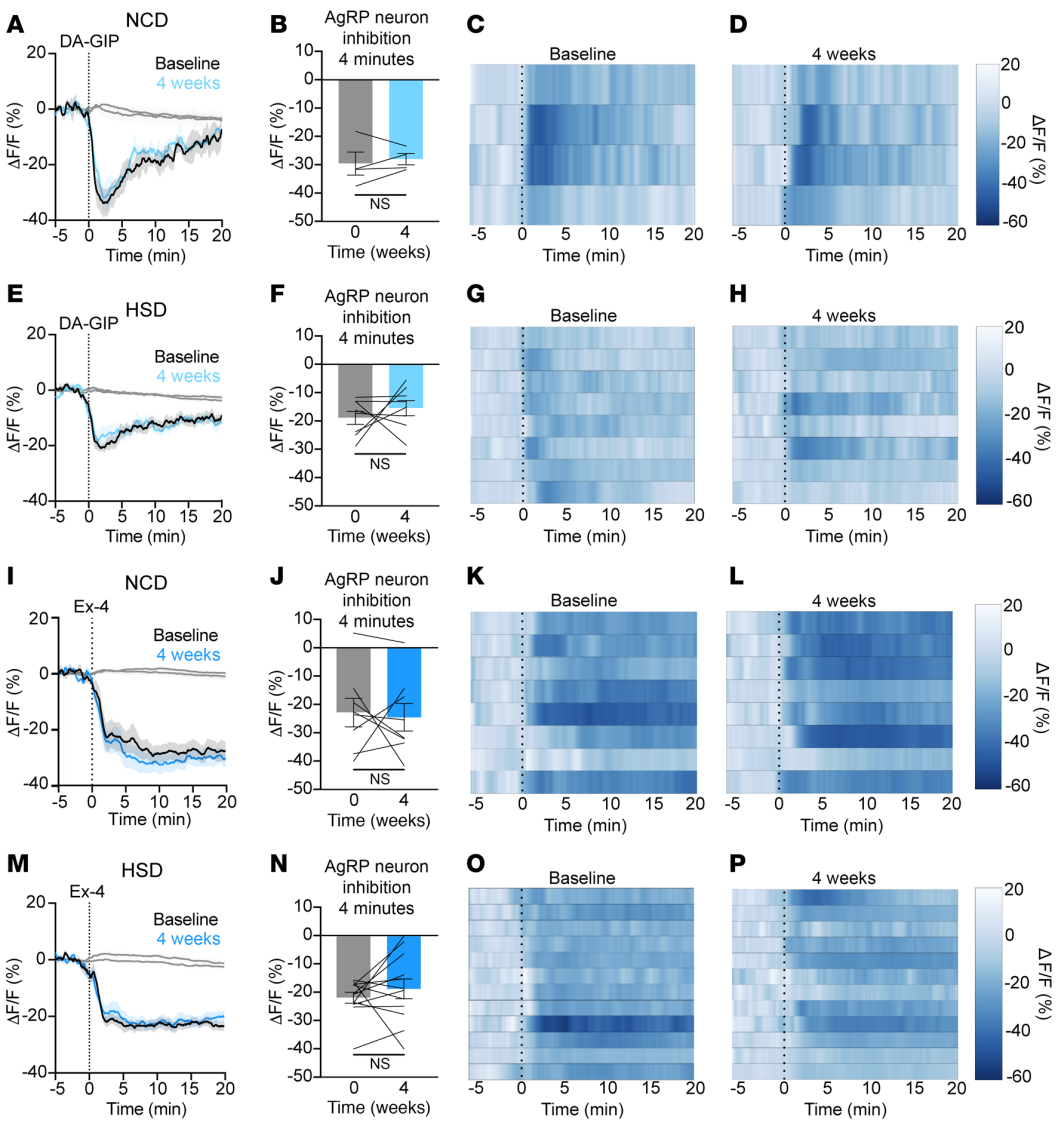
HSD-induced obesity does not alter GIPR or GLP-1R agonist-induced AgRP neuron inhibition. (**A**, **E**, **I**, and **M**) Calcium signal in AgRP neurons from fasted NCD (**A** and **I**) and HSD (**E** and **M**) mice injected with DA-GIP (1 mg/kg; **A** and **E**) or Ex-4 (0.5 mg/kg; **I** and **M**) at baseline and after 4 weeks of chow or HSD (4 weeks) as indicated. *n* = 4–7 mice per group for DA-GIP; *n* = 8–12 mice per group for Ex-4. (**B**, **F**, **J**, and **N**) Average ΔF/F in mice from **A**, **E**, **I**, and **M**) 4 minutes after injection. (**B**) paired *t* test, *P* = 0.5744; (**F**) paired *t* test, *P* = 0.4588; (**J**) paired *t* test, *P* = 0.7781; (**N**) paired *t* test, *P* = 0.3850). (**C**, **D**, **G**, **H**, **K**, **L**, **O**, and **P**) Heatmaps showing ΔF/F in individual mice from **A**, **E**, **I**, and **M** following injection with DA-GIP or Ex-4 at baseline or after 4 weeks on the indicated diet. (**A**, **E**, **I**, and **M**) Isosbestic traces for all recordings are shown in gray. (**A**, **C**–**E**, **G**–**I**, **K**–**M**, **O**, and **P**) Vertical dashed lines indicate the time of injection. (**B**, **F**, **J**, and **N**) Lines represent individual mice. Error bars indicate mean ± SEM.

These findings illuminate roles for incretins in the regulation of AgRP neuron dynamics. However, the acute effects of incretin receptor agonists and antagonists in lean mice may not reflect their actions in diet-induced obese (DIO) mice. We therefore set out to address the effect of incretin receptor agonism on AgRP neuron dynamics in mice over time and to gain insight into the effect of these drugs in DIO mice. To do this, we subjected mice to a recently developed, obesogenic high-sucrose diet (HSD) that attenuates glucose-induced AgRP neuron inhibition over the course of 4 weeks (19). As we previously reported, 4 weeks of HSD leads to weight gain compared with a normal chow diet (NCD) (NCD: [4-week weight] – [baseline weight] = 1.92 g ± 0.32 g; HSD: [4-week weight] – [baseline weight] = 4.49 g ± 0.74 g; *P* = 0.03). However, HSD-induced obesity did not significantly alter AgRP neuron responses to incretin agonists. NCD-fed mice also exhibited consistent responses to incretin agonist injection over this time course (Figure 6). Thus, while multiple studies have shown that obesity alters AgRP neuron responses to food presentation and to gastrointestinal nutrients (17–19), these changes are unlikely to be incretin-mediated, as neural responses to incretin receptor agonists remain intact in obese mice.

## Discussion

### GIP but not GLP-1 is required for nutrient-mediated AgRP neuron inhibition.

Our findings reveal roles for AgRP neurons in in vivo incretin physiology and pharmacology. In particular, the physiologic function of GIP in food intake and body weight maintenance has remained elusive. Numerous studies have shown that GIPR agonism reduces food intake (29, 30). By contrast, other studies have shown that global GIPR-KO mice are protected from both obesity and insulin resistance when fed a high-fat diet (31–35), and GIPR antagonism coupled with GLP-1R agonism leads to weight loss in early clinical trials and mouse models (36, 37), possibly by preventing leptin resistance (16, 38, 39). Here, we have identified a role for endogenous GIP in gut-brain communication. The role of GIP signaling in glucose-mediated AgRP neuron inhibition (Figure 1) is consistent with the anorexigenic effects and obesity treatment efficacy of GIPR agonists. Further studies will be required to define which GIPR-expressing neurons are required to elicit this effect and to determine how both agonism and antagonism of GIPR promote weight loss.

The lack of effect of GLP-1R antagonism on nutrient-mediated AgRP neuron inhibition is an equally important finding in this study (Figure 2). It is in line with multiple prior studies demonstrating that endogenous GLP-1 is not critical for regulating food intake, and that GLP-1R–KO mice and mice treated with GLP-1R antagonists are also protected from obesity (32, 40, 41). Of note, the mechanism underlying the small but significant delayed reduction in chow-mediated AgRP neuron inhibition following GLP-1R blockade is unclear (Supplemental Figure 2, E–G), and the finding puzzling, given that GLP1R blockade does not impact nutrient-mediated AgRP neuron inhibition or feeding. We hypothesize that recently characterized GLP-1R– and TRH-expressing arcuate nucleus neurons that send inhibitory projections to AgRP neurons may mediate this effect (42). This is an important topic of future investigation. Taken together, our current findings indicate that GLP-1 release in response to nutrient intake is not required for rapid modulation of AgRP neuron activity or regulation of feeding.

### GIPR and GLP-1R agonism inhibit AgRP neurons, abrogate their response to food presentation, and may play a role in incretin-mimetic efficacy.

In addition to illuminating a role for endogenous GIP, we have also shown that AgRP neurons may play a role in mediating the anorexigenic effects of pharmacologic incretin receptor agonism. Building off of prior studies showing that a variety of systemically administered, anorexigenic gut-secreted hormones rapidly inhibit AgRP neurons (7, 8), and that stimulation of AgRP neurons can overcome gut hormone–induced anorexia (43), we showed that fast-acting GLP-1 and GIP analogs rapidly inhibit AgRP neurons (Figure 3). Prior studies have shown GLP-1 analogs inhibit AgRP neurons in slice preparations and modulate *Agrp* gene expression (21, 44), but they did not demonstrate an acute in vivo effect of peripherally administered GLP-1R agonist on AgRP neuron dynamics. Optogenetic stimulation of AgRP neurons partially restored food intake following treatment with incretin agonists (Figure 4), suggesting that AgRP neuron inhibition may contribute to the anorexigenic effect of incretin-mimetic therapies. In addition to acutely inhibiting AgRP neurons, pretreatment with DA-GIP, Ex-4, or both, dramatically attenuated food presentation–induced AgRP neuron inhibition in a manner that mirrors the degree of feeding suppression induced by these agonists (Figure 5). By contrast, incretin receptor agonist pretreatment appeared to mildly attenuate the response of AgRP neurons to intragastric nutrients, though this effect did not reach significance (Supplemental Figure 6). Thus, to the extent that AgRP neurons are involved in the appetite-suppressing effects of incretin mimetic therapies, it may be, in part, through reducing food intake driven by anticipatory inhibition rather than altering postingestive gut-brain signaling. Collectively, these findings add to our mechanistic understanding of incretin-based antiobesity agents, but many questions remain to be addressed.

It is unclear what cell types and circuits GLP-1R and GIPR agonists act upon to inhibit AgRP neurons, though based on prior ex vivo physiology studies and RNA-seq findings, this effect is likely indirect (20, 21, 44–46). GLP-1R is expressed in feeding-related nuclei in the hypothalamus and brainstem (47, 48), and knockout from glutamatergic but not GABAergic neurons almost entirely abrogates GLP-1R agonist-induced weight loss in obese mice (49). While hypothalamic or hindbrain knockdown of GLP-1R reduces liraglutide efficacy, no brain region has been shown to be solely responsible for GLP-1R agonist-induced food intake suppression (21, 50–52). GLP-1R is also expressed in a large population of vagal afferent neurons (53–56), and chemogenetic activation of these distension-sensing nodose ganglion neurons is sufficient to inhibit AgRP neurons (55). Moreover, central blockade of GLP-1R does not abrogate the anorexic effects of peripherally administered Ex-4 (57), and GLP-1R deletion from peripheral sensory neurons modestly attenuates the appetite suppressing and weight loss efficacy of GLP-1R agonists in obese mice (58, 59). By contrast, recent work shows that ablation of GLP-1R-expressing nodose ganglion neurons does not blunt GLP-1R agonist-induced weight loss (52). Thus, GLP-1–induced appetite suppression and weight loss may be mediated directly or indirectly by multiple peripheral and central neural circuits (60). Alongside prior studies, our data suggest that AgRP neurons are an indirect but critical target of GLP-1–based therapies.

Similarly, GIPR is expressed in hypothalamic feeding centers, area postrema, and NTS but not in hypothalamic AgRP neurons (45, 46). CNS knockout of the GIPR from GABAergic neurons blocks the modest anorectic effects of long-acting GIPR agonists and abrogates the benefit of dual GLP-1 and GIP receptor agonism when compared with GLP-1R monoagonism in obese mice (30, 45, 61). Chemogenetic activation of GIPR-expressing cells in the hypothalamus or dorsal vagal complex reduces feeding, but local GIPR knockout in the hypothalamus does not blunt incretin-mimetic induced weight loss (45, 62). GIPR is also expressed at low levels in nodose and dorsal root ganglia, but its function in these cell populations has not been examined (53, 62–64). Recent data support a critical role for spinal afferent neurons in glucose-mediated AgRP neuron inhibition (12), and it is possible that GIPR activation in these neurons is involved. Additional studies examining the effect on feeding and neural activity of local, cell-type specific GIPR knockout mice are necessary to clarify the physiologic and pharmacologic roles of this hormone.

### DIO does not alter incretin agonist-induced AgRP neuron inhibition.

Mounting data indicate that DIO blunts gut-brain communication, and that obesogenic diets of differing macronutrient composition have distinct effects on this axis (17–19). Specifically, we recently showed that a HSD selectively dampens glucose-mediated AgRP neuron inhibition without affecting AgRP neuron inhibition induced by other nutrients. Given our current finding that GIPR blockade attenuates glucose-induced AgRP neuron inhibition, we set out to test the hypothesis that HSD-induced obesity would blunt the response of AgRP neurons to DA-GIP. Surprisingly, one month of HSD did not alter AgRP neuron responses to either GIPR or GLP-1R agonism. While AgRP neuron responses to incretins may change after more prolonged or severe obesity, our results indicate that molecular signals other than incretin hormones mediate altered gut-brain dynamics following sucrose overconsumption.

In summary, gut hormone receptor agonism has ushered in a new era of obesity management with the efficacy of multireceptor agonism rivaling that of bariatric surgery. Understanding the molecular and circuit-based mechanisms of hormone-mediated appetite control is critical to refine and more precisely target future therapies to the key cell types mediating the transformative effects of these drugs. Using modern neuroscience and genetic approaches, we have dissected the role of AgRP neurons in incretin-mediated gut-brain communication and elucidated physiologic and pharmacologic effects of GLP-1 and GIP on this axis.

## Methods

### Sex as a biological variable

Experiments were performed in male and female mice 2–6 months of age unless otherwise indicated. Male and female data were combined.

### Animals

Mice were housed in a 12/12-hour reverse light/dark cycle with ad libitum chow (Envigo, 7012, Teklad LM-495 Mouse/Rat Sterilizable Diet) and water access. HSD-fed DIO mice were maintained on ad libitum chow and water, and also had ad libitum access to a 25% w/v sucrose solution for 4 weeks, as recently described (19). Mice were fasted for 5 or 16 hours before experiments, as indicated in the text and figures. During fasting periods, mice had ad libitum water access. *Agrp^tm1(cre)Lowl^* (AgRP-Cre, #012899, Jackson Labs) animals backcrossed onto a C57BL/6J background were used for fiber photometry and nutrient infusion experiments. For optogenetic experiments, AgRP-Cre mice were crossed with B6.Cg-Gt(ROSA)26Sor^tm32(CAG-COP4*H124R/EYFP)Hze^/J mice (ROSA26-loxStoplox-ChR2-eYFP, #024109, Jackson Labs), to generate AgRP:ChR2 animals. C57BL/6J mice (wildtype #000664, Jackson Labs) were used to measure food intake following incretin hormone agonist and antagonist injections. Experiments were performed during the dark cycle in a dark environment.

### Stereotaxic surgery

For photometry experiments, AAV expressing Cre-dependent GCaMP6s (100842-AAV9, AAV9.CAG.Flex.GCaMP6s, Addgene) was injected unilaterally above the arcuate nucleus (ARC) of AgRP-Cre mice. During the same surgery, an optical fiber (MFC_400/430-0.48_6.3mm_MF2.5_FLT, Doric Lenses) was implanted unilaterally at the coordinates x = +0.25 mm, y = –1.65 mm, z = –5.95 mm from bregma. Mice were allowed 2 weeks for recovery and viral expression before beginning experiments or implanting intragastric catheters.

For optogenetic experiments, fiberoptic implants (MFC_200/245_0.37_6.1mm_ZF1.25_FLT, Doric Lenses) were placed unilaterally above the ARC of AgRP:ChR2 mice at the coordinates x = +0.25 mm, y = –1.63 mm, z = –5.85 mm from bregma. Mice were allowed 10 days for recovery, during which they were habituated to handling, intraperitoneal injection, and tethering to patch cords in feeding chambers before performing experiments. Following both surgeries, mice were treated with meloxicam and buprenorphine.

### Intragastric catheter implantation

Surgery was performed as previously described (7, 65). AgRP-Cre mice with working photometry implants were anesthetized with ketamine/xylazine. An incision was made between the scapula, and the skin was dissected from the subcutaneous tissue. An abdominal incision was made from the xiphoid process caudally. A sterilized catheter was pulled into the abdominal cavity via a small puncture in the abdominal wall. The stomach was externalized, punctured, and the catheter was inserted into the puncture site and sutured in place. The stomach was returned to the abdominal cavity and the abdominal muscle and skin were sutured. Lastly, the catheter was secured at its intrascapular cite using a felt button (VABM1B/22, Instech Laboratories), and the intrascapular skin incision was sutured. Postoperatively, mice were treated with meloxicam, buprenorphine, and a dose of enrofloxacin, and allowed 14 days to recover before experiments.

### Fiber photometry

Two photometry processors were used in this study (RZ5P and RZ10X, TDT). For the RZ5P setup, the LEDs and LED driver are separate from the processor (DC4100 (LED driver); M405FP1 and M470F3 (LEDs), Thorlabs), while the RZ10X processor has these components integrated. Each mouse was run on the same system using the same patch cord for every recording session to allow for reliable within-mouse comparisons.

Blue LED (465-470 nm) and UV LED (405 nm) were used as excitation light sources. LEDs were modulated at distinct rates and delivered to a fluorescence minicube (Doric Lenses) before connecting to the mouse implants (MFC_400/430-0.48_6.3mm_MF2.5_FLT, Doric Lenses) via patch cords (MFP_400/430/1100-0.57_2m_FCM-MF2.5_LAF, Doric Lenses). Emissions were collected through the patch cords to photoreceivers (Newport Visible Femtowatt Photoreceiver for the RZ5P system; integrated Lux photosensors in the RZ10X system). Digital signals were demodulated, lock-in amplified, and collected through the processors. Data were collected using Synapse software (TDT).

During recordings, mice were placed in operant chambers (ENV-307W-CT, Med Associates) within light- and sound-attenuating cubicles (ENV-022MD, Med Associates) with no food or water access unless otherwise indicated. Mice with AgRP signals inhibited less than 20% by chow presentation were considered technical failures and excluded from further experiments.

### Nutrient infusions during fiber photometry recording

Nutrients were infused via intragastric catheters using a syringe pump during fiber photometry recordings as previously described (7). All infusions were given at 0.1 mL per minute for 10 minutes for a total volume of 1 mL. All nutrient infusions were calorie matched at 0.5 kcal. Glucose, intralipid and Ensure were dissolved in deionized water. All photometry experiments involving infusions were performed in overnight-fasted AgRP-Cre mice.

To determine whether signaling through GIPR is critical for nutrient-mediated AgRP neuron inhibition, mice equipped for fiber photometry recording and intragastric nutrient infusion were given an injection of a control, nonneutralizing antibody at 30 mg/kg IP (16) (provided by Eli Lilly) and fasted for 16 hours prior to recordings. At the end of the 16-hour fast, the syringe pump was attached to the intragastric catheter using plastic tubing and adaptors, and mice were habituated to the photometry recording chambers for 20 minutes prior to nutrient infusions. Calorie- and volume-matched infusions of glucose, intralipid, or Ensure were given on different days and recording continued for 10 minutes after the end of infusion. These infusions were completed over the course of 7–10 days, and mice were reinjected with the control antibody every 7 days. After completing these infusions, mice were injected with a previously characterized neutralizing mouse anti-murine GIPR antibody (muGIPR-Ab (16), provided by Eli Lilly) at 30 mg/kg and fasted 16 hours before a second round of nutrient infusions was completed as described above. muGIPR-Ab was dosed weekly based on previously published studies (16, 38). AgRP neuron inhibition induced by nutrient infusions was compared across the 2 conditions. The long-lasting effects of GIPR antibody blockade precluded us from balancing treatment order, and thus recordings following muGIPR-Ab were each performed 7–10 days after control recordings. Additionally, given its long-lasting effects, for all mice that received muGIPR-Ab, subsequent nutrient infusion was a final experiment before euthanasia and confirmation of implant placement and viral expression. To control for changes in fiber photometry signal strength over time as a possible cause of muGIPR-Ab effects, a separate cohort of untreated mice were given nutrient infusions at the same time points indicated above without antibody administration.

To determine whether signaling through GLP-1R is critical for nutrient-mediated AgRP neuron inhibition, mice were habituated to the photometry recording chamber for 20 minutes, then pretreated with the GLP-1R antagonist Exendin (9-39) (Ex-9, 1 mg/kg) (HY-P0264, MedChemExpress) or vehicle (saline) 5 minutes prior to infusion of glucose, intralipid, or Ensure on separate days. Neural recordings were continued for 10 minutes after the end of infusions. Neural responses to infusions following Ex-9 versus vehicle pretreatment were measured 7–10 days apart for each nutrient, and treatment order was counterbalanced across mice.

### Hormone injections

Exendin-4 (Ex-4) (HY-13443, MedChemExpress) and (D-Ala^2^)-GIP (DA-GIP) (4054476, Bachem) were injected intraperitoneally (IP) at the doses indicated in the text and figure legends. Where indicated, Ex-4 and DA-GIP were diluted in the same solution and injected simultaneously. All hormones were dissolved in saline. To monitor AgRP neural response to hormone treatment using fiber photometry, AgRP-Cre mice were habituated to handling, photometry recording chambers, and IP injections. For recordings, mice were placed in the chambers for 20 minutes prior to injection. Following injection, the photometry recording continued for 20 minutes. To evaluate the effects of hormones on the response of AgRP neurons to food presentation or gastrointestinal nutrients, we presented mice with chow or delivered an intragastric Ensure infusion 20 minutes after hormone injection.

To evaluate the effects of Ex-4 and DA-GIP on food intake, WT C57BL/6J mice were habituated to handling, IP injection, and individual feeding chambers before undergoing a 5 hour fast at the start of dark cycle. Following the fast, mice received an IP injection of saline, DA-GIP (1 mg/kg), Ex-4 (0.02 mg/kg), or DA-GIP and Ex-4 given simultaneously. Mice were immediately refed in feeding chambers, and food consumption was measured at 4 hours. Each mouse received all treatments on different days and treatment order was counterbalanced.

To evaluate the effect of muGIPR-Ab on food intake, C57BL/6J mice were habituated to handling, IP injection, and individual feeding chambers before undergoing an overnight fast with injection of either control or muGIPR-Ab (30 mg/kg) at the start of fasting. Mice were refed in feeding chambers, and food consumption was measured over 2 hours. Due to the long-lasting effects of muGIPR-Ab, all mice received control antibody first.

To evaluate the effect of Ex-9 on food intake, C57BL/6J mice were habituated to handling, IP injection, and individual feeding chambers before undergoing an overnight fast. Following the fast, mice received an IP injection of saline or Ex-9 (1 mg/kg). Mice were immediately refed in feeding chambers and food consumption was measured over 2 hours. Each mouse received both treatments on different days, and treatment order was counterbalanced.

### Optogenetic feeding experiments

AgRP:ChR2 mice were group housed and ranged from 4 to 12 months old. For 10 days during recovery from surgery, mice were habituated to handling, recording chambers, and patch cord tethering. An LED source and TTL pulse generator (D-OG-LED-B/B, Prizmatix) were used to generate blue light (460 nm, 2 s ON/3 s OFF, 10 ms pulse width, 20 Hz, 10-20 mW at the fiber tip). Fiber optic patch cables (500 μm POF N.A. 0.63 L=75 cm, Prizmatix) were connected to the mouse implants (MFC_200/245-0.37_6.1mm_ZF1.25_FLT, Doric Lenses) via a sleeve (MFC_200/245-0.37_6.1mm_ZF1.25_FLT, Doric Lenses).

On test days, mice were given 30 minutes of habituation without LED stimulation or chow. Following habituation, mice received an IP injection of saline, Ex-4 (0.02 mg/kg), or DA-GIP (1 mg/kg) plus Ex-4 (0.02 mg/kg) simultaneously. After injection, mice were immediately given 30 minutes of access to chow with or without light stimulation. Each experiment was performed in the fasted state (5 hours, beginning at start of dark cycle) in the same mice on different days. Hormone treatment order was counterbalanced.

### Quantification and statistical analysis

#### Photometry analysis.

Photometry data were analyzed with custom Python scripts (https://github.com/nikhayes/fibphoflow; commit ID dde086457be2aa31925fbf7891b86629dd748603), and statistical analyses and data visualizations were generated with Python and Prism. Photometry recordings included emissions from 470 nm stimulation and from 405 nm stimulation, which were smoothed and downsampled to 1 Hz. Normalization of responses to stimuli relative to baseline was performed on each these signals via the formula: ΔF / F = (F_t_ – F_0_) / F_0_, where F_t_ represents fluorescence at time (t), and F_0_ represents the average fluorescence during the 5-minute baseline period preceding the stimulus start time (time 0). To determine statistical significance, the average ΔF / F was calculated for the time frames indicated in the legend for Figures 1–3, 5, and 6, and Supplemental Figures 1–6.

#### Behavioral data analysis.

To determine chow consumption during fast refeeding and optogenetic experiments, chow was weighed manually at the indicated time points.

#### Statistics.

Fiber photometry data were collected and analyzed as previously described (7, 17, 19). For photometry traces shown in Figures 1, 2, 3, 5, and 6, and Supplemental Figures 1–6, ΔF / F (%) refers to the mean ΔF_t_ / F_0_ × 100. For bar graphs quantifying neural responses to chow presentation (Figure 5 and Supplemental Figures 2, 4, and 5), the average ΔF / F over a 1-minute period at the time points indicated in the figures was calculated. For bar graphs quantifying neural responses to nutrient or water infusion (Figures 1 and 2, and Supplemental Figures 1, 3, and 6), the average ΔF / F over a 1-minute period at the end of nutrient infusion (9–10 min) was calculated. For bar graphs quantifying neural responses to hormone injection (Figures 3 and 6, and Supplemental Figures 4 and 5), the average ΔF / F over a 1-minute period at the time points indicated in the figures was calculated.

The effects of experimental manipulation versus controls were analyzed with a 1-way, repeated-measures ANOVA (Figures 3 and 5, and Supplemental Figures 4 and 5) or paired 2-tailed *t* tests (Figures 1, 2, and 6, and Supplemental Figures 1, 2, 3, and 6) as appropriate for photometry experiments. Fast refeeding in WT mice after treatment with saline or incretin agonists (Figure 5) was analyzed with a 1-way, repeated-measures ANOVA. Fast refeeding in WT mice after treatment with saline, control antibody, or incretin receptor antagonists (Supplemental Figure 2) was analyzed with a 2-way, repeated-measures ANOVA. Food intake in the presence or absence of light stimulation following saline or incretin agonist administration (Figure 4) was analyzed with a 2-way, repeated-measures ANOVA. The Holm-Šídák multiple comparisons test was used as appropriate. Prism was used for all statistical analyses, and significance was defined as *P* < 0.05. Sample sizes are indicated in the figure legends for each experiment. Where multiple technical replicates of an experiment were performed, trials from the same animal were averaged and handled as a single biological replicate for data analysis and visualization.

### Study approval

Experimental protocols were approved by the Northwestern University IACUC in accordance with NIH guidelines for the Care and Use of Laboratory Animals.

### Data availability

Custom Python scripts used in this manuscript have been deposited in Github: (https://github.com/nikhayes/fibphoflow). Values for all data points in graphs are reported in the Supporting Data Values file. All raw data files will be made available upon request to the corresponding author.

## Author contributions

HEM designed experiments, conducted experiments, analyzed data, and wrote the manuscript. ABC, CML, NWH, SWF, JAF, JLX conducted experiments and analyzed data. LRB designed experiments, analyzed data, and wrote the manuscript. RJS designed experiments, provided reagents, and wrote the manuscript.

## Funding support

This work is the result of NIH funding, in whole or in part, and is subject to the NIH Public Access Policy. Through acceptance of this federal funding, the NIH has been given a right to make the work publicly available in PubMed Central.

- American Diabetes Association Pathway to Stop Diabetes Award (12-22-ACE-31).
- NIH grants (P30-DK020595), (K08-DK118188), and (R01-DK128477) (LRB).

## Supplementary Material

Supplemental data

Supporting data values

## Version 1 08/26/2025

In-Press Preview

## Version 2 11/03/2025

Electronic publication
