# Supplementary material for: Incretin receptor agonism rapidly inhibits AgRP neurons to suppress food intake in mice
Source: J Clin Invest. 2025 Aug 26;135(21):e186652. doi: 10.1172/JCI186652 (PMC12578400; doi:10.1172/JCI186652)
Supplement: Supplemental data [file jci-135-186652-s122.pdf]

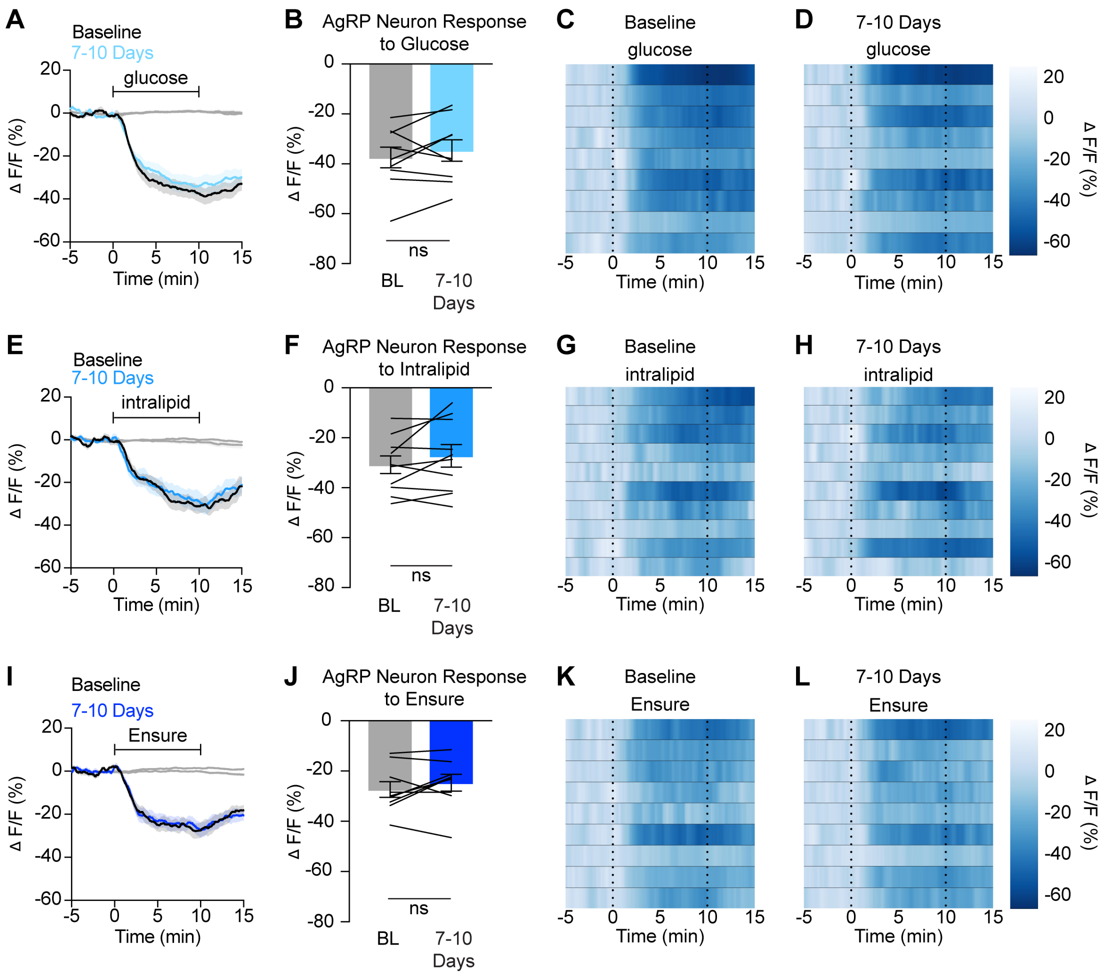

Supplemental Figure 1. Nutrient-mediated AgRP neuron inhibition is stable over time in untreated mice

(A,E,I) Calcium signal in AgRP neurons from fasted mice during infusion of glucose **(A)**, intralipid **(E)**, and Ensure **(I)** at baseline and 7-10 days later as indicated. n = 9-10 mice per group.

(B,F,J) Average ΔF/F in mice from **(A,E,I)** at the end of nutrient infusion. (**(B)** paired t-test, p=0.3471; **(F)** paired t-test, p=0.1781; **(J)** paired t-test, p=0.2725).

(C,D,G,H,K,L) Heat maps showing ΔF/F in individual mice from **(A,E,I)** during nutrient infusion.

(A,E,I) Isosbestic traces for all recordings are shown in gray. **(C,D,G,H,K,L)** Vertical dashed lines indicate the start and end of nutrient infusions. **(B,F,J)** Lines represent individual mice. Error bars indicate mean ± SEM.

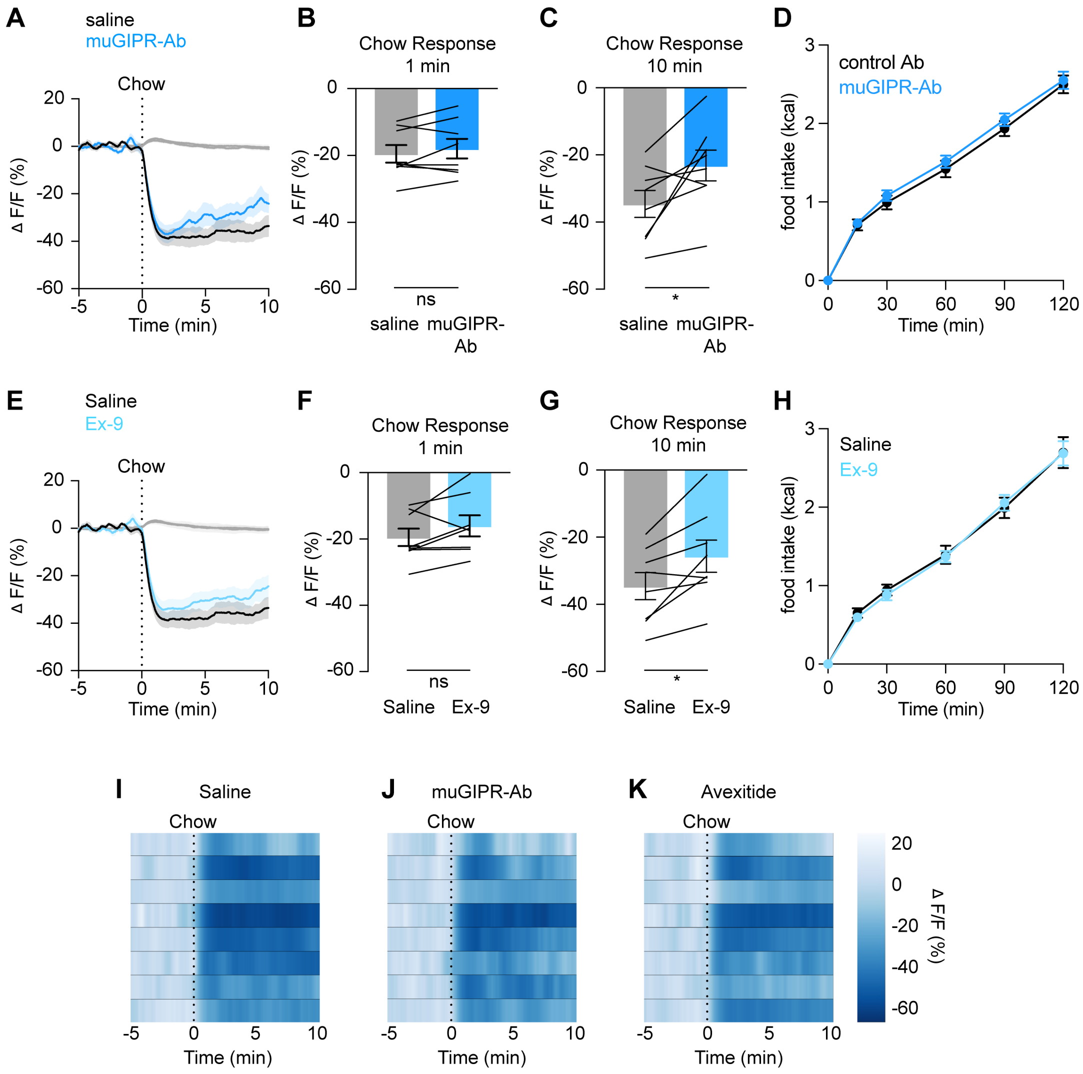

Supplemental Figure 2. GIPR and GLP-1R blockade cause delayed attenuation of the AgRP neural response to chow

(A,E) Calcium signal in AgRP neurons from fasted mice presented with chow after pre-treatment with muGIPR-Ab **(A)**, Ex-9 **(E)**, or saline as indicated. n = 8 mice per group.

(B,C,F,G) Average ΔF/F in mice from **(A,E)** 1 minute **(B,F)** and 10 minutes **(C,G)** after chow presentation. (**(B)** paired t-test, p=0.2629; **(C)** paired t-test, p=0.0318; **(F)** paired t-test, p=0.1270; **(G)** paired t-test, p=0.0111).

(D,H) Two-hour chow intake following an overnight fast and pre-treatment wiith muGIPR-Ab versus control Ab at the time of fasting **(D)** or Ex-9 versus saline immediately before re-feeding **(H)** as indicated in C57BL/6 mice. n = 16 mice per group. (**(D)** two-way ANOVA treatment x time interaction, p=0.8420; **(H)** two-way ANOVA treatment x time interaction, p=0.7569).

(I,J,K) Heat maps showing ΔF/F in individual mice from **(A,E)** after chow presentation.

(A,E) Isosbestic traces for all recordings are shown in gray. **(A,E,I,J,K)** Vertical dashed lines indicate chow presentation. **(B,C,F,G)** Lines represent individual mice. Error bars indicate mean ± SEM. T-tests: *p<0.05.

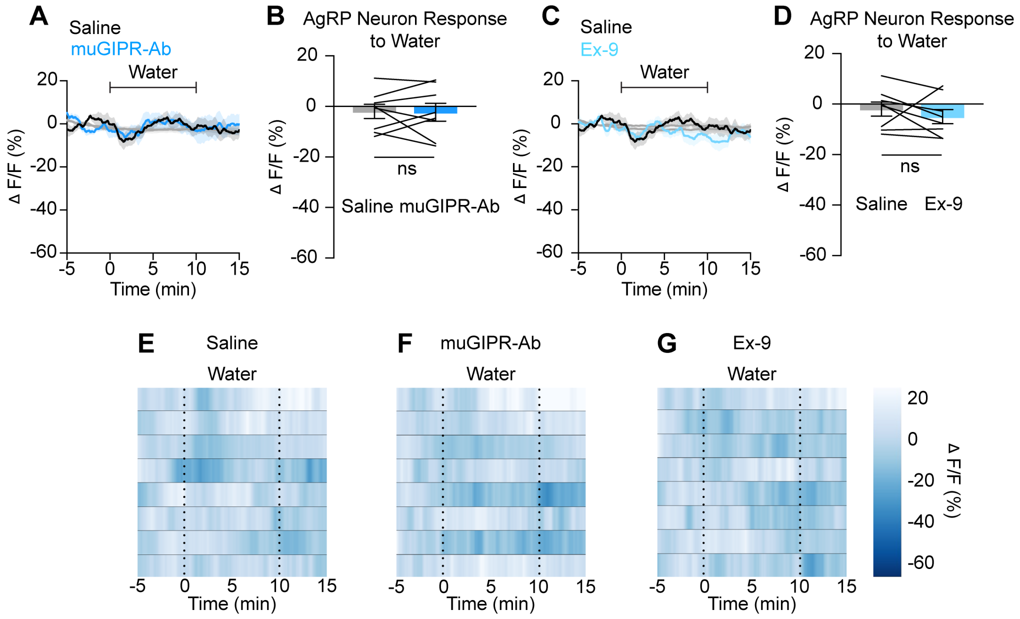

Supplemental Figure 3. GIPR and GLP-1R blockade do not alter AgRP neural response to water infusion

(A,C) Calcium signal in AgRP neurons from fasted mice during water infusion after pre-treatment with muGIPR-Ab **(A)**, Ex-9 **(C)** or saline as indicated. n = 8 mice per group.

(B,D) Average ΔF/F in mice from **(A,C)** at the end of water infusion. (**(B)** paired t-test, p=0.9004; **(D)** paired t-test, p=0.3644).

(E,F,G) Heat maps showing ΔF/F in individual mice from **(A,C)** during water infusion.

(A,C) Isosbestic traces for all recordings are shown in gray. **(E,F,G)** Vertical dashed lines indicate start and end of water infusions. **(B,D)** Lines represent individual mice. Error bars indicate mean ± SEM.

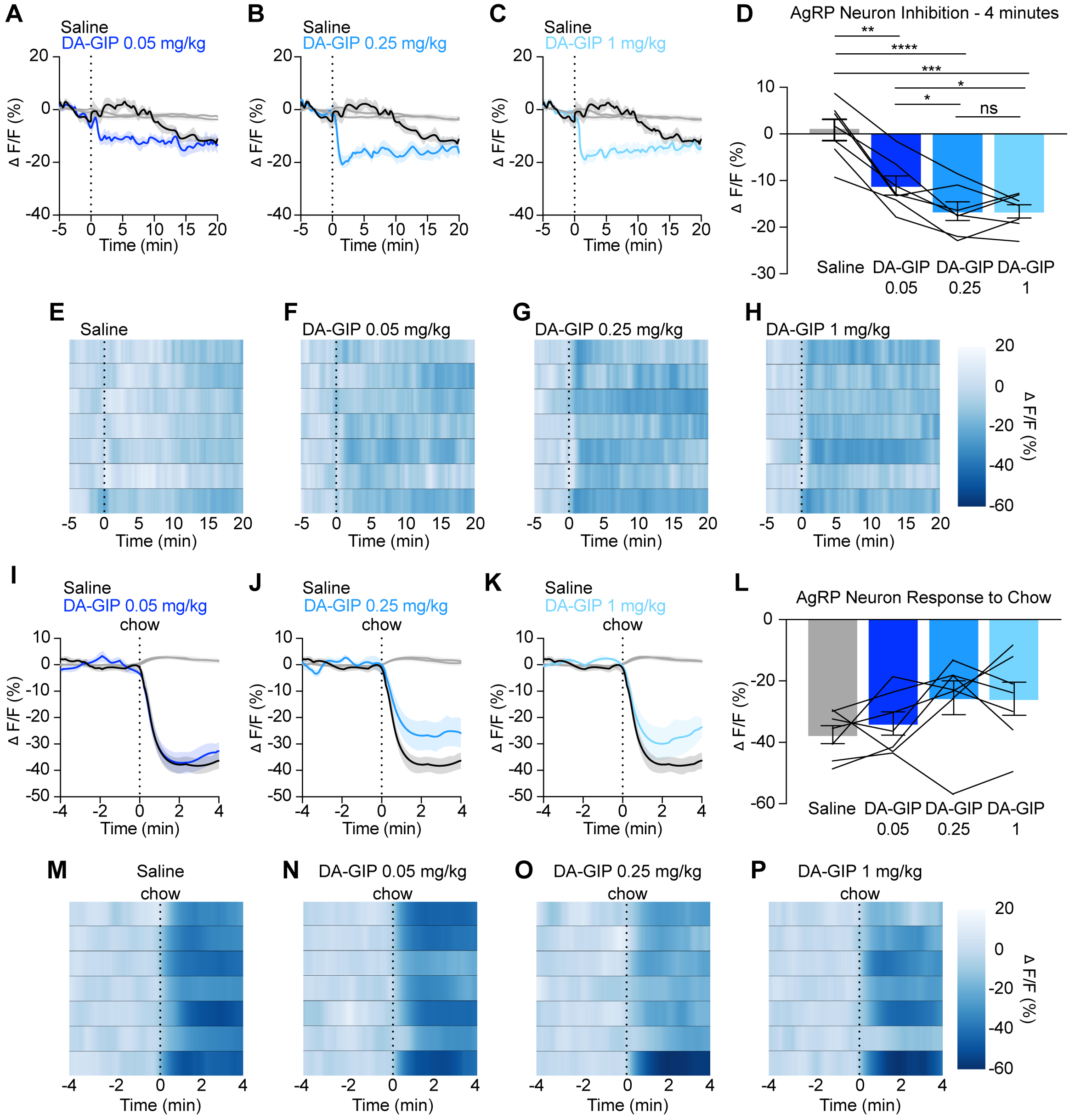

Supplemental Figure 4. AgRP neuron responses to GIPR agonists are dose-dependent
(A-C) Calcium signal in AgRP neurons from fasted mice injected with DA-GIP at 0.05 mg/kg **(A)**, 0.25 mg/kg **(B)**, or 1 mg/kg **(C)** compared to saline as indicated. n = 7 mice per group.
(D) Average ΔF/F in mice from **(A-C)** 4 minutes after injection. (one-way ANOVA, p=<0.0001).
(E-H) Heat maps showing ΔF/F in individual mice from **(A-C)** injected with saline **(E)**, DA-GIP at 0.05 mg/kg **(F)**, 0.25 mg/kg **(G)**, or 1 mg/kg **(H)** as indicated.
(I-K) Calcium signal in AgRP neurons from fasted mice presented with chow 20 minutes after pre-treatment with saline or DA-GIP at 0.05 mg/kg **(I)**, 0.25 mg/kg **(J)**, or 1 mg/kg **(K)** as indicated. n = 7 mice per group.
(L) Average ΔF/F in mice from **(I-K)** 4 minutes after chow presentation. (one-way ANOVA, p=0.0738).
(M-P) Heat maps showing ΔF/F in individual mice from **(I-K)** after chow presentation.
(A-C, I-K) Isosbestic traces for all recordings are shown in gray. **(A-C, E-H, I-K, M-P)** Vertical dashed lines indicate the time of injection or chow presentation. **(D,L)** Lines represent individual mice. Error bars indicate mean ± SEM. Post-hoc comparisons: *p<0.05, **p<0.01, ***p<0.001, ****p<0.0001.

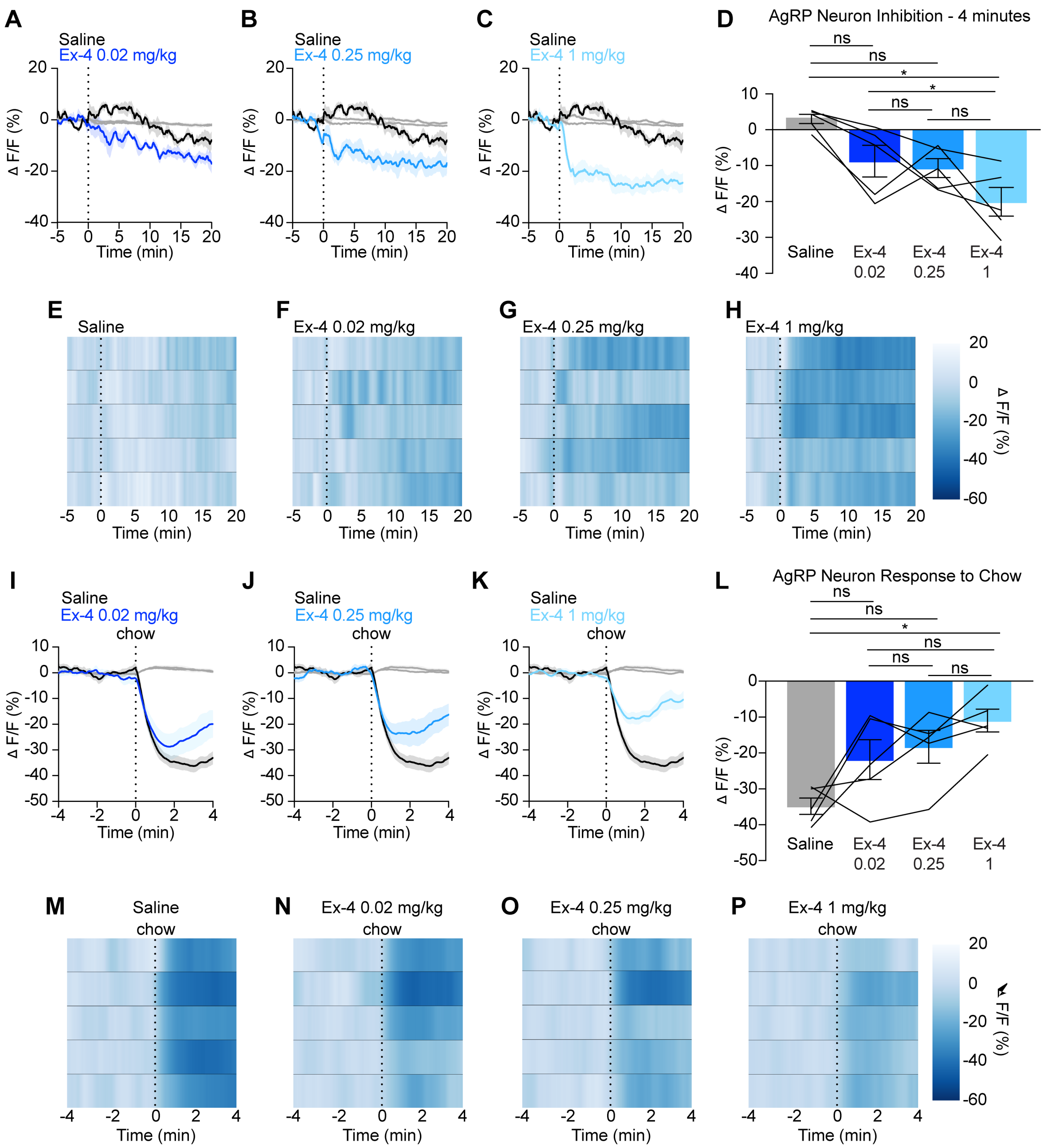

Supplemental Figure 5. AgRP neuron responses to GLP-1R agonists are dose-dependent
(A-C) Calcium signal in AgRP neurons from fasted mice injected with Ex-4 at 0.02 mg/kg **(A)**, 0.25 mg/kg **(B)**, or 1 mg/kg **(C)** compared to saline as indicated. n = 5 mice per group.
(D) Average ΔF/F in mice from **(A-C)** 4 minutes after injection. (one-way ANOVA, p=0.0119).
(E-H) Heat maps showing ΔF/F in individual mice from **(A-C)** injected with saline **(E)**, Ex-4 at 0.02 mg/kg **(F)**, 0.25 mg/kg **(G)**, or 1 mg/kg **(H)** as indicated.
(I-K) Calcium signal in AgRP neurons from fasted mice presented with chow 20 minutes after pre-treatment with saline or Ex-4 at 0.02 mg/kg **(I)**, 0.25 mg/kg **(J)**, or 1 mg/kg **(K)** as indicated. n = 5 mice per group.
(L) Average ΔF/F in mice from **(I-K)** 4 minutes after chow presentation. (one-way ANOVA, p=0.0219).
(M-P) Heat maps showing ΔF/F in individual mice from **(I-K)** after chow presentation.
(A-C, I-K) Isosbestic traces for all recordings are shown in gray. **(A-C, E-H, I-K, M-P)** Vertical dashed lines indicate the time of injection or chow presentation. **(D,L)** Lines represent individual mice. Error bars indicate mean ± SEM. Post-hoc comparisons: *p<0.05.

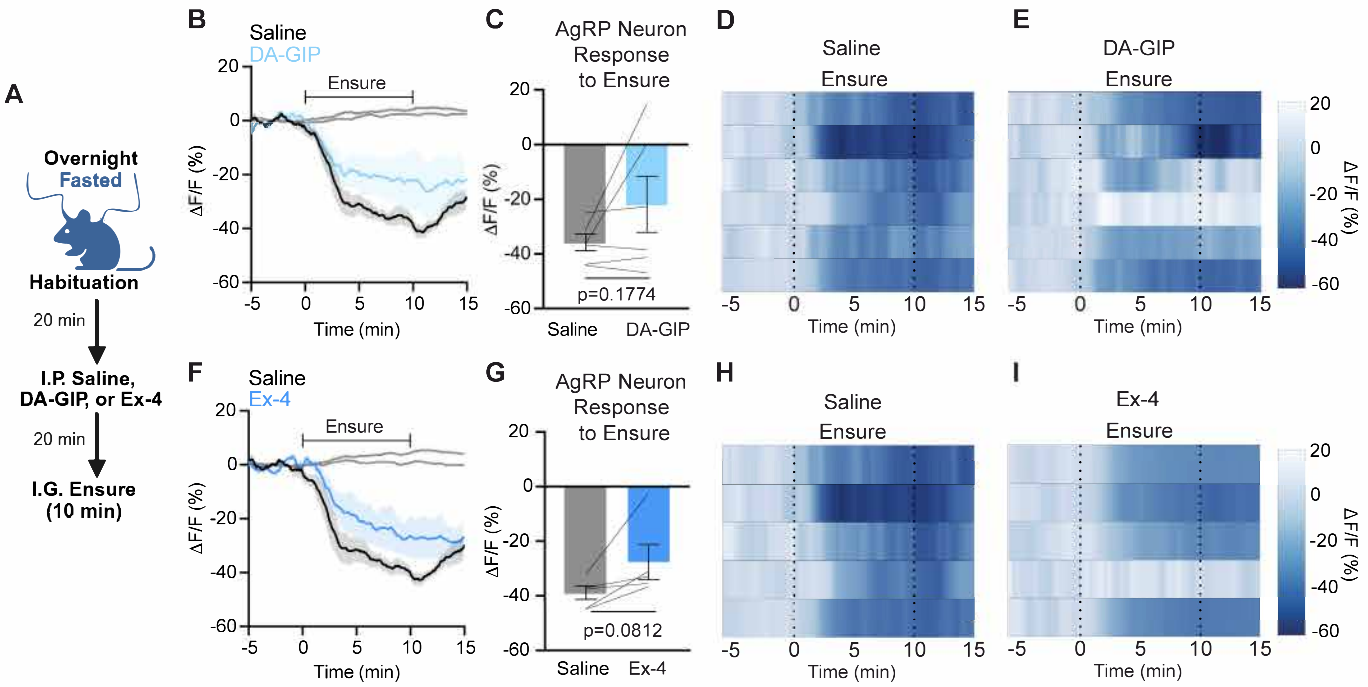

Supplemental Figure 6. GIPR and GLP-1R agonists do not attenuate intragastric nutrient-mediated AgRP neuron inhibition

(A) Experimental schematic.

(B,F) Calcium signal in AgRP neurons from fasted mice during intragastric infusion of Ensure 20 minutes after pre-treatment with saline, DA-GIP **(**1 mg/kg; **B)**, or Ex-4 **(**0.5 mg/kg; **F)** as indicated. n = 5-6 mice per group.

(C,G) Average ΔF/F in mice from **(B, F)** at the end of Ensure infusion. (**(C)** paired t-test, p = 0.1774; **(G)** paired t-test, p = 0.0812).

(D,E,H,I) Heatmaps showing ΔF/F in individual mice from **(B, F)** during Ensure infusion after pre-treatment with saline **(D, H)**, DA-GIP **(E)**, or Ex-4 **(I)**.

(B,F) Isosbestic traces for all recordings are shown in gray. **(C,G)** Lines represent individual mice. **(D,E,H,I)** Vertical dashed lines indicate the start and end of Ensure infusion. Error bars indicate mean ± SEM.
